# Supplementary material for: Prognostic Value of Lymphoid Infiltration and Aggregation in Cervical Cancer
Source: Cancers (Basel). 2025 Dec 30;18(1):129. doi: 10.3390/cancers18010129 (PMC12785114; doi:10.3390/cancers18010129)
Supplement: Supplementary file 1 [file cancers-18-00129-s001.zip › cancers-4036751-supplementary.pdf]

cancers

MDPI

supplementary materials

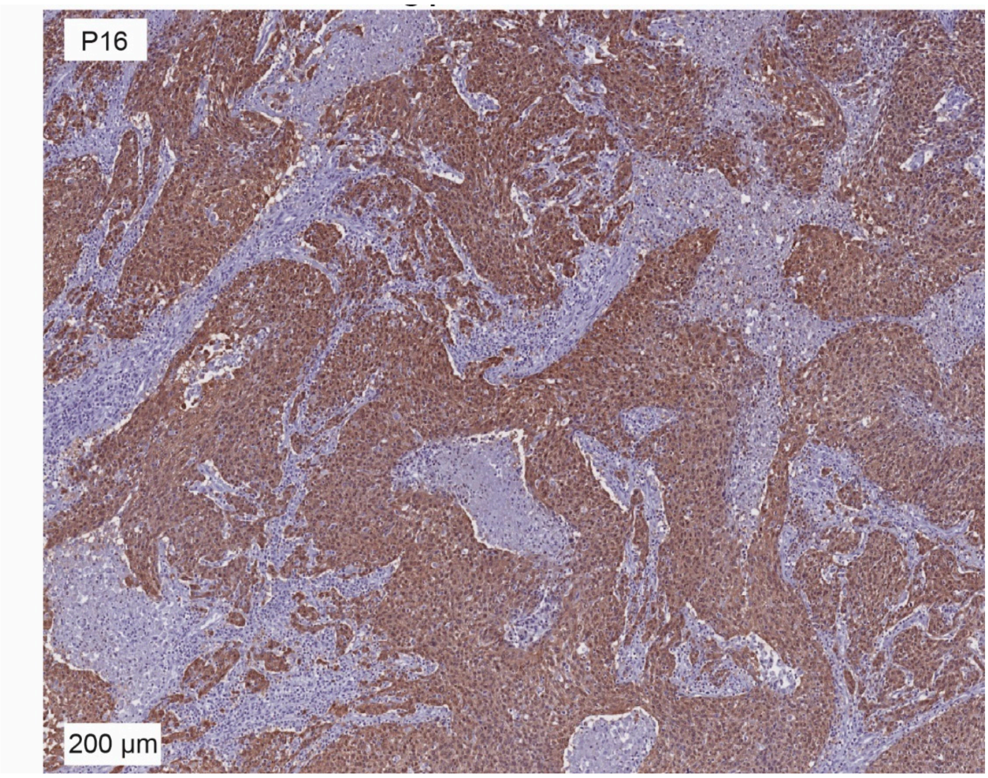

Figure S1. representative P16 IHC staining as a surrogate for HPV status.

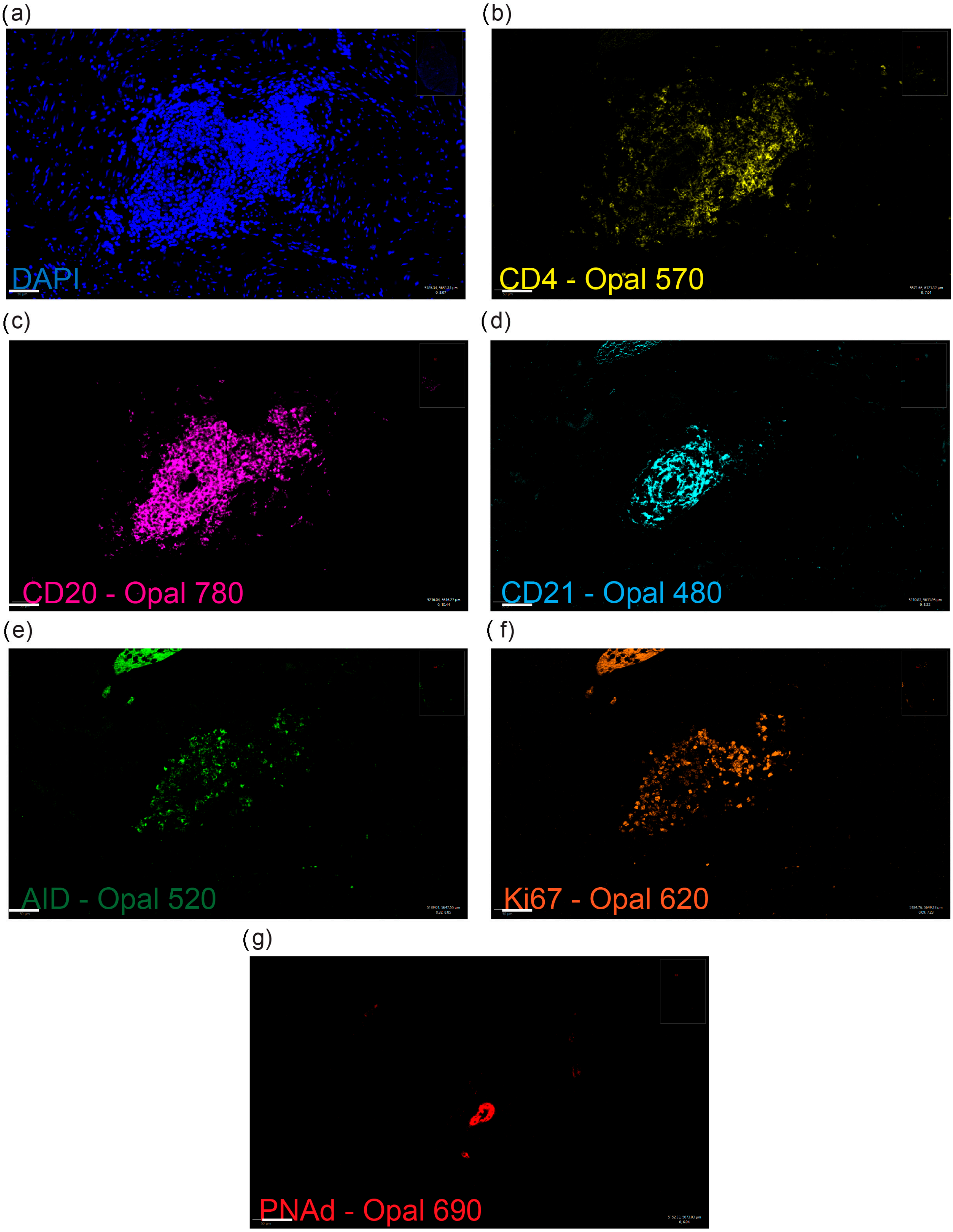
(a)
DAPI
(b)
CD4 - Opal 570
(c)
CD20 - Opal 780
(d)
CD21 - Opal 480
(e)
AID - Opal 520
(f)
Ki67 - Opal 620
(g)
PNAd - Opal 690

Figure S2. Multiplex immunofluorescent stained "Activity Panel" shown in single channel view. (**a**) DAPI (**b**) Opal 570 – CD4 (**c**) Opal 780 – CD20 (**d**) Opal 480 – CD21 (**e**) Opal 520 – AID (**f**) Opal 620 – Ki67 (**g**) Opal 690 – PNAd. Scale bar= 50um.

Table S1. Hazard Ratios for univariate overall survival (OS).

**Univariate Overall Survival**			
	HR	95% CI	*P*-value
Lymphoid Aggregate	2.067	[5.827–0.733]	0.170
Cxcl13	0.421	[1.238–0.143]	0.116
Cd8	0.288	[0.909–0.091]	0.034
Depth of Invasion	1.096	[1.177–1.020]	0.012
Tumor Size	1.363	[1.884–0.987]	0.060
Age	1.042	[1.098–0.989]	0.123
Histology	0.658	[1.818–0.238]	0.420
Smoking	0.930	[1.818–0.476]	0.832
Stage	1.461	[2.417–0.883]	0.140
Lvsi	0.948	[2.620–0.343]	0.918

Table S2. Hazard Ratios for multivariate OS.

**Multivariate Overall Survival**			
	HR	95% CI	*P*-value
Lymphoid Aggregate	3.404	[13.304–0.871]	0.078
Cxcl13	0.079	[0.408–0.015]	0.002
Cd8	0.172	[0.735–0.040]	0.018
Depth of Invasion	1.125	[1.348–1.024]	0.002
Tumor Size	1.246	[1.970–0.789]	0.346
Age	0.983	[1.051–0.920]	0.621
Histology	0.877	[1.226–0.628]	0.442

Table S3. Hazard Ratios for univariate recurrence free survival (RFS).

**Univariate Recurrence Free Survival**			
	HR	95% CI	*p*-value
Lymphoid Aggregate	0.968	[2.606–0.360]	0.949
Cxcl13	0.274	[0.853–0.088]	0.025
Cd8	0.370	[1.067–0.128]	0.066
Depth of Invasion	1.030	[1.100–0.964]	0.383
Tumor Size	0.870	[1.213–0.625]	0.412
Age	1.013	[1.070–0.959]	0.636
Histology	1.017	[2.804–0.369]	0.974
Smoking	0.933	[1.825–0.477]	0.839
Stage	1.267	[2.175–0.738]	0.390
Lvsi	0.975	[2.629–0.362]	0.960

Table S4. Hazard Ratios multivariate RFS.

**Multivariate Recurrence Free Survival**			
	HR	95% CI	*p*-value
Lymphoid Aggregate	1.158	[3.908–0.343]	0.813
Cxcl13	0.181	[0.726–0.045]	0.016
Cd8	0.370	[1.296–0.106]	0.120

https://doi.org/10.3390/cancers18010129

Depth of Invasion	1.129	[1.262–1.010]	0.033
Tumor Size	0.900	[1.302–0.622]	0.575
Age	0.991	[1.067–0.921]	0.810
Histology	0.564	[0.971–0.328]	0.039
